# Role of empathy in the perception of medical errors in patient encounters: a preliminary study

**DOI:** 10.1186/s13104-019-4365-2

**Published:** 2019-06-10

**Authors:** Jean Hannan, Gabriel Sanchez, Erica D. Musser, Melissa Ward-Petersen, Elizabeth Azutillo, Deana Goldin, Edgar Garcia Lara, Aniuska M. Luna, Igor Galynker, Adriana Foster

**Affiliations:** 1 Nicole Wertheim College of Nursing and Health Sciences, Florida International University, 11200 SW 8th Street, Academic Health Center 3 Office 324A, Miami, FL 33199 USA; 2 Citrus Health Network/Florida International University, 4175 West 20th Ave, Hialeah, FL 33012 USA; 3 Center for Children and Families, 11200 SW 8th Street, AHC 4, Room 455, Miami, FL 33199 USA; 4 Department of Epidemiology, Florida International University, Robert Stempel College of Public Health & Social Work, 11200 SW 8th Street, AHC5-483, Miami, FL 33199 USA; 5 Nicole Wertheim College of Nursing and Health Sciences, Florida International University, 11200 SW 8th Street, AHC 3 Office 228, Miami, FL 33199 USA; 6 Miami Dade College Benjamin Leon School of Nursing, 950 NW 20th Street, Miami, FL 33127 USA; 7 Department of Psychiatry & Behavioral Health, Herbert Wertheim College of Medicine, Florida International University, 11200 SW 8th Street, AHC4 280, Miami, FL 33199 USA; 8 Icahn School of Medicine, Beth Israel–Fierman, 317 E 17th St, New York, NY 10003 USA; 9 Department of Psychiatry & Behavioral Health, Herbert Wertheim College of Medicine, Florida International University, 11200 SW 8th Street, AHC1 335A, Miami, FL 33199 USA

**Keywords:** Empathy, Medical error, Adherence, CARE measure, Communication

## Abstract

**Objective:**

Healthcare professionals’ empathy have been empirically demonstrated to decrease the risk of medical errors. Medical errors affect patient’s outcomes and healthcare providers’ well-being. Therefore, the purpose of this study was to determine the relationship between patients’ perception of healthcare providers’ empathy, their intention to adhere to treatment, and their perception of medical errors made. An anonymous survey was emailed to staff at a health center and an urban university in Miami, Florida, USA.

**Results:**

A total of 181 participants were enrolled. Participants rating their healthcare provider as high in empathy had 80% lower odds of reporting errors (CI 0.04–0.6). The intention to follow-up with recommendations or return to the provider were not significantly associated with provider’s empathy. Patients of high empathy providers were no more treatment adherent that those who rated their provider with low empathy but were less likely to perceive medical error. Providers’ empathy significantly affected patients’ perception of medical errors. Our results underscore that healthcare curricula need to address the link between empathy and perception of medical errors, including its potential legal implications.

## Introduction

Medical errors, defined as “failure of a planned action to be completed as intended, or the use of a wrong plan to achieve an aim,” [1, 2] were estimated to contribute substantially to mortality in the United States [3]. Medical errors result in high individual and societal costs including lost quality of life, work productivity, and additional medical costs that amount to losses into the billions of dollars [4]. Yet medical errors are preventable. One way to reduce them is by enhancing perceived empathy in patient-healthcare provider interaction. Empathy in healthcare includes understanding the patient’s perspective, communicating that understanding verbally and non-verbally, and acting therapeutically on that understanding [5]. Patients consider empathy to be very important in consultations, and show better treatment adherence and greater satisfaction with more empathetic doctors, while physicians’ communication skills are associated with reduced risk of malpractice claims [5–7]. Levinson et al. [6] called the combination of a bad outcome and patient dissatisfaction, “a recipe for litigation”. In face of a negative treatment result, a provider who relates to a patient in a “negative” manner (i.e. being perceived as less professional, caring, friendly, trustworthy) faces a higher risk of malpractice claims than a provider perceived to relate in a positive manner [8, 9]. Similarly, primary care providers who spend more time with the patient facilitate patient’s involvement in their own care and use humor, face significantly less malpractice claims than providers who show poorer communication skills [6]. While healthcare training programs teach empathy early in their curricula [10–13], longitudinal reinforcement and assessment of this core communication skill varies. Furthermore, empathy, patients’ perception of medical errors and prevention of litigation are addressed separately within the framework of healthcare professions’ competencies [14, 15].

Considering these knowledge gaps and the potential benefits of enhancing patient-healthcare provider interactions through empathy, we investigated whether an association exists between patients’ perception of healthcare providers’ empathy, adherence to medical treatment and perception of medical errors. Our hypotheses were: (1) providers who demonstrate greater empathy elicit greater treatment adherence from their patients, and (2) patients perceive more empathetic providers as making less medical errors than providers who demonstrate lower empathy.

## Main text

### Methods

An anonymous online survey created with Qualtrics software^®^ was disseminated by email to a convenience sample of undergraduate and graduate Florida International University (FIU) nursing students, undergraduate FIU psychology students and FIU employees in Miami, Florida after IRB approval from FIU. The FIU IRB exempted the study waiving the need to obtain an informed consent by the participants being that data was collected anonymously using an online survey.

The same survey was disseminated to healthcare professionals, staff and trainees (psychiatry and psychology residents and psychology postdoctoral students) at a Federally Qualified Health Center, Citrus Health Network in Hialeah, Florida. The study took place in March and April 2018. FIU Institutional Review Board approved the study.

To elicit the patients’ perception of providers’ empathy, we utilized the Consultation and Relational Empathy (CARE) measure [16]. CARE is a validated 10-items instrument that measures patients’ perception of physician’s empathy in the medical encounter. Each item is measured on a 5-point scale (Poor, Fair, Good, Very Good and Excellent). CARE minimum score is 10 and maximum is 50. Normative data on CARE measure identify high-empathy (CARE score 10–30), middle-empathy (score 31–40) and low-empathy providers (score 41–50) [17, 18]. Lower CARE scores of primary care physicians have been associated with poorer patient outcomes [17, 18] and have improved after educational interventions targeting empathy [19].

The CARE measure was preceded by a prompt asking the participants to recall their last health care encounter and questions on whether they had followed through with advice would return to that provider, and if they thought, the provider made any medical errors. A definition of medical error (Kohen [1] “a failure in the process of care that could have been prevented”) was provided in the prompt. The CARE measure was followed by a section requesting demographic background, income bracket and health insurance coverage status. A final prompt allowed participants to submit comments about their experiences (“If you would like to add further comments on this consultation, please do so here”).

We examined descriptive statistics (frequency distributions for categorical variables, mean and standard deviation for continuous variables) for demographics and the outcome variables of interest (follow through with recommendations, intention to return and perception of medical errors). Based on the empathy rating of their last healthcare provider encounter using the CARE measure, survey participants were placed into high, middle and low empathy groups [18]. In order to identify potential covariates, bivariate analyses (using either Chi square tests or independent two sample t-tests) were carried out to examine associations between provider empathy group, survey participant demographics and the outcome variables. After identifying covariates, we used multivariable binary logistic regression models to estimate odds ratios with 95% confidence intervals. We estimated separately the odds the survey participants’: (1) follow-through with provider’s recommendations, (2) intention to return and (3) perception of a medical error. The empathy score group was the main exposure of interest in all models, with the low-empathy provider group considered the reference group. We used Stata 14 software for all analysis.

### Results

Of 195 survey participants, only 181 had complete data on CARE empathy score, gender, and income and thus were included in the final models. Demographic information is summarized in Table 1. Final adjusted models controlled for gender and income.

**Table 1:** Demographics (n = 181)

	Frequency	Percent
Gender		
Male	40	22.10
Female	141	77.90
Race		
White	127	70.17
Black	17	9.39
Other	28	15.47
Unknown	9	4.97
Ethnicity		
Hispanic/Latino	102	56.35
Other	69	38.12
Unknown	10	5.52
Income (USD)		
0–49,400	56	30.94
49,401–127,550	85	46.96
> 127,550	40	22.10
Insurance		
Private Health Insurance	165	91.16
Medicaid/Medicare	8	4.42
VA Care	1	0.55
Uninsured	6	3.31
Other	1	0.55

	Mean	SD
Age^a^	33.51	12.12

^a^Age only available for 175 of 181 respondents

The mean CARE survey score among our participants was 38.94 (SD 9.73), as illustrated in Table 2. To establish whether the low, middle and high empathy scores correlated with perceived error and patient adherence by survey participants, we performed logistic regression analysis and calculated odds ratios. Participants who rated their provider as having high empathy, as compared to those who rated them as having low empathy, had 80% lower odds of reporting medical error (CI 0.04–0.6) and had 220% higher odds of follow through with providers recommendations (CI 0.8–5.8). The latter finding was not statistically significant at a 95% confidence interval, but it is noteworthy because it approaches significance. Those who perceived their physician as showing “middle” empathy score showed no statistically significant difference in odds of either following through with providers’ recommendations or reporting medical error compared to those who rated their provider as having low empathy (Table 3). Regarding likelihood to return to provider, very few participants rated empathy as medium or high and said they would not return to the provider (Table 2).

**Table 2:** Distribution of patients’ perception of healthcare providers’ empathy

	N = 181 subjects	Percentage
CARE score categories		
Low	43	23.76
Middle	43	24.76
High	95	52.49
Follow recommendations		
No	31	17.13
Yes	150	82.87
Return to provider		
No	14	7.73
Yes	167	92.27
Provider made error		
No	166	91.71
Yes	15	8.29

**Table 3:** Association between physicians’ empathy, patient adherence and patient perception of medical errors

	Follow recommendations	Return to provider	Errors in care
	Adjusted OR (95% CI)^a^		Adjusted OR (95% CI)
CARE score^b^			
Low (10–30)	Used as reference		
Middle (31–40)	1.5 (0.5–4.3)	7.0 (1.3–37.5)	0.5 (0.1–1.8)
High (41–50)	2.2 (0.8–5.8)	15.3 (2.9–80.7)	0.2 (0.04–0.6)

Empathy categories defined by Steinhausen et al. [18]

*OR* odds ratio, *CI* confidence interval (95%)

^a^Adjusted models controlled for gender and income

^b^CARE

### Discussion

The mean CARE survey score among our participants was 38.94 with a standard deviation of 9.73, consistent with a meta-analysis of CARE survey results, which found a mean of 40.48 [20]. Our data indicates that patients who perceived their healthcare providers as having high empathy, as compared to those who rated them as having low empathy, had significantly lower odds of reporting medical error.

Patient dis/satisfaction, willingness to follow medical advice and compliance, and perception of empathy were reflected in the scarce descriptive statements provided at the end of the survey. For instance, a respondent who stated: *“I recently changed physicians and I can honestly say that this physician surprised me. He truly took the time to listen to my concerns amidst the other patients he had waiting. I did not feel like I was simply another patient, but a unique individual. I believe that this is a rare quality to find in physicians since they tend to be overwhelmed with work”*, also answered that s/he would go back to said physician, would follow up on his recommendations, and perceived him to not have committed medical errors. The remaining answers in the participant’s survey rated all aspects of their interaction as “excellent”.

Approach-based limitations of the study included the one-time, cross-sectional assessment of the relationship between the survey participants and their healthcare providers. In addition, we did not elicit information about the type of healthcare providers rated by our survey participants, nor did we elicit the duration since the participants’ last healthcare encounter. Our patient sample was small, contained mostly white, female, Hispanic participants and the inquiry was retrospective. Of those who rated their providers’ empathy as medium or high very few said they would not return to the provider, thus leading to wide confidence intervals. Finally, although they offered a few comments, the survey participants did not provide enough details to help characterize medical errors and experiences of empathy linked to their encounters in a significant manner.

### Conclusion

On our relatively small sample of survey participants representing a diverse population in a densely populated urban area, physicians’ empathy significantly affected patients’ perception of medical errors, validating prior work [6, 8, 9] but it did not affect treatment adherence. These results must be replicated in larger studies. The results underscore not only the need of teaching and reinforcing empathy throughout healthcare professions’ curricula, but also the immediate need to focus training on the link between empathy, perception of medical errors and their potential legal and quality of care implications.

### Practice implications

In this study physicians’ empathy significantly affected patients’ perception of medical errors, suggesting that empathy may be inversely related to the potential legal implications of un-empathic care (i.e. malpractice lawsuits). Therefore, teaching empathy as part of medical errors prevention programs throughout healthcare professions curricula and post-licensure may improve quality of life for patients and healthcare providers and prevent the personal, social and economic burden associated with errors.

## Limitations

- A relatively small sample.
- Limited to one area geographically.

## Data Availability

All data generated or analyzed during this study are included in this published article.

## Abbreviations

CARE: Consultation and Relational Empathy
FIU: Florida International University
