# Correction to: Role of empathy in the perception of medical errors in patient encounters: a preliminary study

**DOI:** 10.1186/s13104-019-4518-3

**Published:** 2019-08-13

**Authors:** Jean Hannan, Gabriel Sanchez, Erica D. Musser, Melissa Ward-Peterson, Elizabeth Azutillo, Deana Goldin, Edgar Garcia Lara, Aniuska M. Luna, Igor Galynker, Adriana Foster

**Affiliations:** 1 Nicole Wertheim College of Nursing and Health Sciences, Florida International University, 11200 SW 8th Street, Academic Health Center 3 Office 324A, Miami, FL 33199 USA; 2 Citrus Health Network/Florida International University, 4175 West 20th Ave, Hialeah, FL 33012 USA; 3 Center for Children and Families, 11200 SW 8th Street, AHC 4, Room 455, Miami, FL 33199 USA; 4 Department of Epidemiology, Florida International University, Robert Stempel College of Public Health & Social Work, 11200 SW 8th Street, AHC5-483, Miami, FL 33199 USA; 5 Nicole Wertheim College of Nursing and Health Sciences, Florida International University, 11200 SW 8th Street, AHC 3 Office 228, Miami, FL 33199 USA; 6 Miami Dade College Benjamin Leon School of Nursing, 950 NW 20th Street, Miami, FL 33127 USA; 7 Department of Psychiatry & Behavioral Health, Herbert Wertheim College of Medicine, Florida International University, 11200 SW 8th Street, AHC4 280, Miami, FL 33199 USA; 8 Icahn School of Medicine, Beth Israel–Fierman, 317 E 17th St, New York, NY 10003 USA; 9 Department of Psychiatry & Behavioral Health, Herbert Wertheim College of Medicine, Florida International University, 11200 SW 8th Street, AHC1 335A, Miami, FL 33199 USA

## Correction to: BMC Res Notes (2019) 12:327 10.1186/s13104-019-4365-2

In the original publication of this article [1], an author name was misspelled.

Melissa Ward-Petersen’s name should be corrected to Melissa Ward-Peterson.

The correct name has been included in the author list of this Correction article and has already been corrected in the original article.
